# Phylogenetic and functional trait‐based community assembly within Pacific *Cyrtandra* (Gesneriaceae): Evidence for clustering at multiple spatial scales

**DOI:** 10.1002/ece3.10048

**Published:** 2023-05-04

**Authors:** Melissa A. Johnson

**Affiliations:** ^1^ Daniel K. Inouye US Pacific Basin Agricultural Research Center Hawaii Hilo USA; ^2^ Rancho Santa Ana Botanic Garden CA Claremont USA

**Keywords:** competitive exclusion, environmental filtering, functional traits, islands, neutral processes, niche partitioning

## Abstract

Tropical rainforest communities are often characterized by a small number of species‐rich genera that contribute disproportionately to the alpha diversity in these habitats. In the Pacific Basin, there are nearly 200 species of *Cyrtandra*, most of which are white‐flowered woody shrubs that are single‐island endemics. Within these island communities, multiple *Cyrtandra* species are commonly observed to occur sympatrically in wet forest understories, forming swarms of what appear to be ecologically similar taxa. The aim of this study was to determine whether species of these plants are randomly assembled with respect to phylogenetic relatedness and traits that are ecologically relevant. I examined assembly patterns across three Pacific archipelagoes using a combination of 10 functional traits and a well‐resolved phylogeny comprising 34 species of *Cyrtandra*. Coexisting species were found to be more closely related and more phenotypically similar than would be expected by chance. This pattern was observed at both regional (island) and local (site) spatial scales. The retention of phylogenetic signal in floral traits and the strong influence of these traits on the observed degree of phylogenetic clustering may indicate that generalist insect pollinators act as a biotic filter on oceanic islands, driving selection for similar floral morphology among closely related species of Pacific *Cyrtandra*. Phylogenetic signal was also detected in leaf size, which contributed to niche clustering at both spatial scales. Coupled with a propensity for long‐distance dispersal, and the restricted distribution of *Cyrtandra* to rainforest understories, this finding suggests that environmental filtering along this trait axis may be more important than dispersal limitation in determining species assemblages. This study supports the theory that plant species are not randomly assembled, and instead, that niche‐based processes structure biodiversity at regional and local spatial scales in diverse congeneric species assemblages.

## Graphical abstract

In the Pacific Basin, multiple *Cyrtandra* species are commonly observed to occur sympatrically in wet forest understories, forming swarms of what appear to be ecologically similar taxa. The aim of this study was to determine whether species of these plants are randomly assembled with respect to phylogenetic relatedness and traits that are ecologically relevant. The retention of phylogenetic signal in floral traits and the strong influence of these traits on the observed degree of phylogenetic clustering may indicate that pollinators act as a biotic filter for closely related species of *Cyrtandra*.

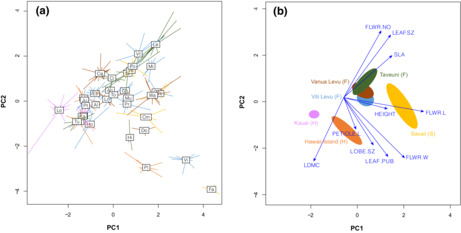

## 1 INTRODUCTION

A fundamental question in ecology and evolutionary biology is how particular species come together to form communities. Three major processes have been put forth to explain the structuring of species assemblages. Under the process of competition, species with shared niches are expected to compete for resources, such that one species will eventually exclude the other, or selective pressure will result in character displacement. A pattern of even spacing or overdispersion in traits among coexisting species is often interpreted as evidence for competition's role in community assembly (Dayan & Simberloff, 2005; Schluter, 2000). In contrast, biotic and abiotic conditions can create a filter, such that species with similar ecological requirements are found in comparable habitats (Cornwell et al., 2006; Weiher & Keddy, 1999). Species may be ecologically similar due to shared evolutionary history, or the independent evolution of similar traits (i.e., trait convergence). These two processes thus result in opposing patterns, with competition resulting in niche partitioning and habitat filtering resulting in niche clustering. A third explanation for community assembly patterns involves neutral processes, under which species abundances result from a combination of dispersal, speciation, and stochastic variation in birth and death rates (i.e., ecological drift). Under neutral theory, the presence or absence of a given species in a community is dependent on chance events, not on the ability of the species to compete (Hubbell, 2001). However, neutral processes can be difficult to detect as a combination of biotic interactions and environmental filtering may produce seemingly stochastic, or neutral, patterns (Purves & Pacala, 2005). Ultimately, species coexistence may depend on how habitat filtering, competition, and neutral processes interact over ecological and evolutionary timescales (Webb et al., 2002).

Additionally, the relative importance of processes involved in community assembly likely varies depending on the spatial scale examined (Cavender‐Bares et al., 2006; Emerson & Gillespie, 2008; Swenson et al., 2007). At regional scales, environmental filtering is hypothesized to structure species assemblages, as higher habitat heterogeneity and availability allows closely related species with similar niche requirements to successfully establish, resulting in phylogenetic clustering (Cavender‐Bares et al., 2009; Swenson et al., 2007; Weiher & Keddy, 1995). In contrast, at local spatial scales habitats should be more homogeneous, thereby leading to increased interspecific competition. This would limit the coexistence of close relatives and result in patterns of phylogenetic evenness or overdispersion (Bryant et al., 2008). However, this reasoning follows the assumption that phylogenetic similarity reflects similarity in ecologically relevant traits, which is not always true (Cahill et al., 2008; Losos, 2008).

A combination of ecologically relevant trait data and an understanding of the phylogenetic history of species may provide the most satisfactory approach for elucidating patterns of community assembly (Kraft & Ackerly, 2010; Kraft et al., 2007; Sedio et al., 2012). In the tropics, most studies that have employed these methods have focused on describing community assembly patterns within particularly diverse rainforest regions such as the Amazon (Baraloto et al., 2012; Kraft et al., 2008; Lebrija‐Trejos et al., 2010). While such studies have greatly advanced our understanding of community assembly patterns and processes, the high taxonomic diversity at this scale can rarely be assessed below the family or genus level given present‐day gaps in taxonomic knowledge. Furthermore, with few genic regions sequenced across many plant families and/or genera, phylogenetic relationships at this scale are often poorly resolved. This combination of issues has resulted in some uncertainty regarding the inferred patterns of community assembly in plants.

Fewer studies have sought to understand assembly patterns within species‐rich plant genera that host multiple sympatric taxa across their distributional range (but see *Banksia* sensu Merwin et al., 2012; *Psychotria* sensu Sedio et al., 2012; *Piper* sensu Salazar et al., 2016; *Inga* sensu Dexter et al., 2017). Such studies stand to provide unique insight into the problem of community assembly, in that congeneric species have had a relatively short period of time in which to diverge from their close relatives, compared with species belonging to different families or genera. Congeneric species may be able to coexist by partitioning niche space via divergence in key functional traits. However, studies that have examined assemblage patterns within a single genus often suffer from the same limitations as large‐scale studies of community assembly, with weakly supported phylogenies constructed from one or two genic regions (as in Merwin et al., 2012; Salazar et al., 2016; Sedio et al., 2012), and/or data for only one or a few functional traits (as in Merwin et al., 2012; Salazar et al., 2016).

The traits selected to examine patterns of plant community assembly have traditionally been vegetative features that have some bearing on the physiological functioning of plants (reviewed in E‐Vojtko et al., 2020). In contrast, few studies have examined traits that are indirectly involved in competition for biotic resources (but see Alcantara et al., 2014; Sargent & Ackerly, 2008; Wolowski et al., 2017). Vegetative and floral traits are subject to differing selective forces, and the decoupling of these two trait groups across species can provide insights into how plants are able to coexist (E‐Vojtko et al., 2022). Differentiation in species floral and vegetative traits may allow for coexisting taxa to utilize the same resources by employing alternative modes of acquisition. Niche segregation via floral traits may occur through pollination (Kay et al., 2005; Kay & Sargent, 2009), florivory (Boaventura et al., 2022), and/or hydrological niches (Roddy et al., 2021). Given that floral traits have been found to play a significant role in the diversification of angiosperms, the inclusion of these traits in studies of plant community assembly is warranted.

The genus *Cyrtandra* J.R. & G. Forster (Gesneriaceae) is a diverse group of plants, with ca. 800 species distributed across Southeast Asia and the Pacific (Atkins et al., 2013). Centers of diversity for the group include Borneo (ca. 200 spp.), the Philippines (ca. 150 spp.), and New Guinea (ca. 120 spp.). The remote volcanic islands of the Pacific are also exceptionally species‐rich, with ca. 175 *Cyrtandra* species distributed across an area that extends from the Solomon Islands, east to the Marquesas Islands, and north to the Hawaiian Islands (Atkins et al., 2013). *Cyrtandra* is estimated to have emerged ca. 16 million years ago in Borneo, followed by widespread dispersal (likely by avian frugivores) and diversification of the crown group ca. 13 million years ago across Southeast Asia (Atkins et al., 2020). The genus followed a west‐to‐east dispersal route from New Guinea to the Solomon Islands and then to Fiji; Fiji and Samoa served as the main source of dispersal into the remote Pacific with all major crown lineages emerging within the last 5 million years (Johnson et al., 2017). Plants of species of *Cyrtandra* are morphologically diverse in habit (small trees, shrubs, or vines), flower color (pink, red, purple, yellow, green, and white), and fruit type (indehiscent capsule or fleshy berry). However, plants of Pacific species of *Cyrtandra* are predominantly small trees or shrubs with white flowers and white fleshy fruits. Species are restricted to the understory of montane to lowland rainforests and occasionally mesic valleys, although several species occur near sea level.

*Cyrtandra* is an ideal study system for understanding patterns of species coexistence within a single genus, as anywhere from 2 to 13 sympatric species have been observed to occur within a single community (Gillett, 1967; Wagner et al., 1990; M. Johnson, pers. obs.). Bramley et al. (2004) examined community assembly of *Cyrtandra* on Mount Kerinci, Sumatra, where 13 species are known to coexist. The authors used a phylogeny based on the ITS region to elucidate the timing of speciation events (ancient vs. recent) and evolutionary origin (repeated colonization vs. in situ radiation) of *Cyrtandr*a taxa at a local scale. This study found evidence for three distinct lineages, which showed uneven rates of diversification, and were likely older than the volcano itself, with only one lineage exhibiting more recent speciation. A second study investigating community assembly in Hawaii Island *Cyrtandra* using nuclear SNPs also found evidence for multiple colonization events and uneven diversification, as well as hybridization between colonizing lineages (Johnson et al., 2019). While these studies improved our understanding of community assembly processes in *Cyrtandra*, the inferences that could be made were limited by the spatial scale at which sampling was conducted (local or regional) and by the source of data (phylogenetic only).

In this study, I sought to expand on these earlier studies by examining the influence of whole plant, vegetative, and floral functional traits on community assembly patterns at both regional and local spatial scales within Pacific *Cyrtandra* using a well‐resolved phylogeny based on nuclear and chloroplast data. The aims of this study were to address the following questions: (1) Are communities of Pacific *Cyrtandra* randomly assembled with respect to relatedness? (2) Is there evidence of overdispersion or clustering with respect to ecologically relevant traits? (3) Do trait‐based patterns of community assembly reflect evolutionary relatedness? Findings from this study stand to provide insight into the processes that govern community assembly of closely related species at different spatial scales and provide new perspectives on the evolution of this hyperspecies‐rich genus across the Pacific Basin.

## 2 MATERIALS AND METHODS

### 2.1 Study species and sites

During the months of June to August 2013–2016, plants were sampled in their native habitat across three Pacific Island archipelagos that host high numbers of endemic *Cyrtandra* species: Fiji (42 spp.), Samoa (19 spp.), and the Hawaiian Islands (60 spp.). Islands within archipelagos were selected to encompass a range of substrate ages, land area, and isolation from source areas (Table S1). Sites within islands were selected to encompass the range of habitats (based on differences in elevation, climate, and vegetation type) occupied by Pacific *Cyrtandra* species, as well as a range of numbers of sympatric species (Table 1). Given the remote location and limited accessibility of many sites, it was not possible to set up permanent plots in which to explicitly test coexistence at varying spatial scales. Instead, plants were sampled along belt‐like transects that extended 1 km in length and 2 m in width. Transects followed trails through the forest understory (either preestablished or established during this study) or topographic features such as creeks, valleys, or ridgelines. GPS points were taken along each transect, and site localities were classified into specific habitat types according to those described in Mueller‐Dombois and Fosberg (1998).

**TABLE 1:** Site information for sampled communities of *Cyrtandra* in the Pacific, listed by increasing elevation. Species in bold were collected from the specified site for the present study; species in black were included in analyses for the specified site due to previous observations of the species at the site; species in gray were previously observed for that site but were not included in the present study due to a lack of phylogenetic and/or functional trait data.

Study site	Island (country)	Elevation (m)	Habitat type	Species
Lavena	Taveuni (FJ)	20–33	Lowland rainforest	***C. gregoryi***, ***C. tempestii***
Komave	Viti Levu (FJ)	50–55	Lowland rainforest	***C. involucrata***, ***C. aloisiana***
Waisali Village	Vanua Levu (FJ)	110–130	Lowland rainforest	***C. longifructosa***
Navua River	Viti Levu (FJ)	125–130	Lowland rainforest	***C. milnei***, ***C. vitiensis***, ***C. hornei***, *C. anthropophagorum*, *C. trichophylla*
Colo‐i‐Suva	Viti Levu (FJ)	150–185	Lowland rainforest	***C. cephalophora***, ***C. esothrix***, ***C. vitiensis***, ***C. pritchardii***, ***C. tomentosa***, *C. anthropophagorum*, *C. milnei*
Nambukelevu	Viti Levu (FJ)	165–180	Lowland rainforest	***C. involucrata***, ***C. leucantha***, *C. muskarimba*
Mt. Korobaba	Viti Levu (FJ)	240–260	Lowland rainforest	***C. esothrix***, ***C. milnei***, ***C. pritchardii***, *C. tomentosa*, *C. anthropophagorum*, *C. vitiensis*, *C. cephalophora*, *C. trichophylla*
Mt. Voma	Viti Levu (FJ)	270–290	Lowland rainforest	***C. anthropophagorum***, ***C. vitiensis***, *C. coleoides*, *C. milnei*, *C. hornei*, *C. multiseptata*
Waisali Forest Reserve	Vanua Levu (FJ)	335–355	Lowland rainforest	***C. cephalophora***, ***C. dolichocarpa***, ***C. waisaliensis***
Mt. Naitaradamu	Viti Levu (FJ)	400–445	Lowland rainforest	***C. anthropophagorum***, ***C. coleoides***, ***C. milnei***, ***C. multiseptata***, ***C. vitiensis***, *C. tomentosa*, *C. esothrix*, *C. involucrata*, *C. hornei*, *C. cephalophora*, *C. acutangula*, *C. trichophylla*, *C. montana*
Taga	Savaii (WS)	470–600	Lowland rainforest	***C. pogonantha***, ***C. richii***
Mt. Koroyanitu	Viti Levu (FJ)	630–680	Montane mesic forest	***C. hornei***, *C. milnei*, *C. involucrata*, *C. vitiensis*
Matavanu Crater	Savaii (WS)	675–705	Montane rainforest	***C. compressa***, ***C. falcifolia***, ***C. richii***, *C. pogonantha*, *C. nudiflora*
A‘opo	Savaii (WS)	765–875	Montane rainforest	***C. compressa***, ***C. richii***, *C. aurantiicarpa*, *C. nudiflora*
Waiakea Forest Reserve	Hawaii Island (HI)	960–990	Montane rainforest	***C. lysiosepala***, ***C. paludosa***, ***C. platyphylla***, *C. giffardii*
Mt. Lomalagi	Viti Levu (FJ)	1020–1090	Cloud forest	***C. hornei***, ***C. multiseptata***, ***C. prattii***, ***C. vitiensis***, *C. jugalis*, *C. victoria*, *C. leucantha*
Des Voeux Peak	Taveuni (FJ)	975–1150	Cloud forest	***C. ciliata***, ***C. hispida***, ***C. leucantha***, ***C. tuiwawai***
Koke‘e State Park	Kauai (HI)	1215–1260	Montane mesic forest	***C. kauaiensis***, ***C. longifolia***, *C. paludosa*
Mt. Tomanivi	Viti Levu (FJ)	1000–1270	Cloud forest	***C. chlorantha***, ***C. victoriae***, ***C. vitiensis***, ***C. jugalis***, *C. coleoides*, *C. esothrix*, *C. milnei*, *C. involucrata*, *C. multiseptata*, *C. prattii*, *C. occulta*

Treatments of the genus *Cyrtandra* in Fiji (Gillett, 1967), Samoa (Gillett, 1973), and the Hawaiian Islands (Wagner et al., 1990) were initially used to assign individuals to species. Identities were later confirmed using a combination of phylogenetic data and morphological comparisons with herbarium specimens. In total, 25 species were sampled from three islands in Fiji (Viti Levu, Vanua Levu, and Taveuni), four species were sampled from one island in Samoa (Savaiʻi), and five species were sampled from two of the Hawaiian Islands (Kauaʻi and Hawaiʻi Island). Voucher specimens (deposited at RSA, SUVA, and PTBG) and silica‐dried leaf material for phylogenetic analyses were collected from multiple individuals per species at each site. When possible, widespread species were sampled from multiple populations across the range of their known distribution, whereas species with smaller ranges were often sampled from a single population.

To determine community membership at each field site, distribution information was first compiled for each sampled species from herbarium specimens and from treatments of *Cyrtandra* in Fiji (Gillett, 1967), Samoa (Gillett, 1973), and Hawaii (Wagner et al., 1990). For each site that was sampled, the author included: (1) all species that were collected from the site during my field surveys for this study, and (2) all species previously collected at the site (according to herbarium specimens) but that were not seen during my field surveys. For this second group of species, the author was able to acquire samples from nearby sites (see Table 1). In this way, the author attempted to capture the full suite of species more accurately in each community, some of which may not have been detected during my field surveys since many species are present in low densities.

### 2.2 Functional traits

For each species, 1–25 individuals were sampled for functional trait measurements depending on how common the species was at each site. A range of whole plant, leaf, and floral traits were selected to capture potential differences in abiotic resource use and biotic interactions that may affect species coexistence (Table 2). Protocols for measuring whole plant and leaf functional traits follow those of Cornelissen et al. (2003). The height (cm) of individual plants was measured from the base to the crown with an extendable meter stick. For leaf traits, two replicate leaves per individual were sampled at the third node from the crown. Leaves at this position were fully mature, yet not senescing. Leaves with herbivore damage were avoided. Petiole length, leaf length, and leaf width (at the widest part of the leaf) were measured in cm. The petiole was then removed from the leaf blade, and the leaves were immediately weighed with a portable scale in the field to acquire fresh leaf mass (g). Digital photographs were taken of each leaf with a ruler for scale to calculate leaf area in ImageJ (Abramoff et al., 2004). Leaf pubescence was also assessed in ImageJ by using the cell counter plug‐in to estimate the number of trichomes in a cm^2^ on the adaxial leaf surface. Leaves were then dried to a constant mass in a 60° oven to acquire dry leaf weight (g). Specific leaf area (SLA, m^2^ kg^−1^) was calculated by dividing leaf area by fresh leaf mass, and leaf dry matter content (LDMC, mg g^−1^) was calculated by dividing dry leaf mass by fresh leaf mass.

**TABLE 2:** Description of functional traits, abbreviations, units of measurement, and inferred ecological relevance.

Functional trait	Abbreviation	Unit	Ecological relevance
Maximum height	Height	cm	Competitive vigor, fecundity, light capture
Specific leaf area	SLA	m^2^ kg^−1^	Photosynthetic rate, leaf life span
Leaf dry matter content	LDMC	mg g^−1^	Photosynthetic rate, leaf life span, growth rate, water balance
Petiole length	Petiole.L	cm	Light capture efficiency
Leaf pubescence	Leaf. Pub	cm^2^	Water balance, herbivore protection
Leaf size	Leaf.Sz	cm^2^	Leaf cooling, light capture
Corolla tube length	Flwr.L	mm	Pollinator selection
Corolla tube width	Flwr.W	mm	Pollinator selection
Corolla lobe size	Lobe.Sz	mm^2^	Pollinator attraction
Flower number	Flwr.No	#	Pollinator attraction

For floral traits, one to two flowers were measured per plant depending on the availability of open flowers. Given that *Cyrtandra* species are protandrous, only flowers in the female (ovulate) phase were used for measurements, as flowers attain their maximum size at this time. Calipers were used to measure the length (mm) and width (at the mouth, in mm) of the corolla tube, as well as the length (mm) and width (at the widest part, in mm) of the posterior corolla lobe. To assess the size of floral displays, the maximum number of flowers per cymose inflorescence was counted. For each trait in which subsampling was performed, the replicate measurements for each plant were averaged to get a mean value for each individual. Four species from the island of Viti Levu in Fiji were not reproductive at the time of field surveys (*C. aloisiana*, *C. jugalis*, *C. pritchardii*, and *C. tomentosa*). For these species, values for flower number per inflorescence were taken from Gillett's (1967) taxonomic treatment, and flower length, width, and lobe size were estimated from herbarium specimens.

A principal components analysis (PCA) was conducted to reduce dimensionality of the trait data and evaluate the functional phenotype of each species. The relationship between species and functional traits was examined by fitting traits onto the ordination space using the function *envfit* in the vegan package (Oksanen et al., 2015) for the R statistical environment (R Development Core Team, 2014). This multivariate correlation analysis partitions the linear component of each predictor on the final PCA axes.

### 2.3 Phylogeny reconstruction

**FIGURE 1:**
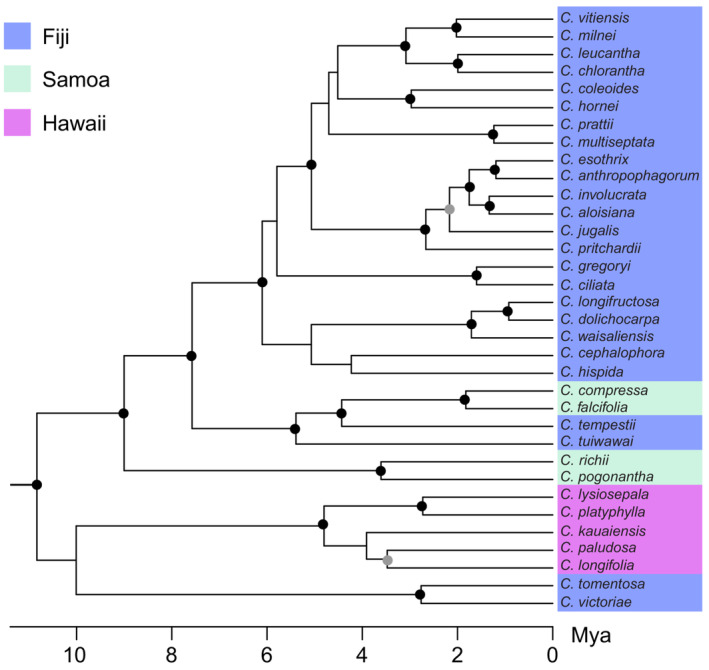
Species phylogeny of 34 Pacific *Cyrtandra* from Fiji, Samoa, and the Hawaiian Islands. Filled black circles indicate support values of ≥70 BS (maximum likelihood bootstrap support) and ≥0.95 PP (Bayesian posterior probability), while gray circles indicate support values of ≥70 BS or ≥0.95 PP.

The time‐calibrated phylogeny of Johnson et al. (2017) was used to infer relationships among Pacific *Cyrtandra* species. This phylogeny includes 109 *Cyrtandra* taxa sampled across three nuclear (ITS, ETS, and Cyrt1) and two chloroplast loci (*rpl*32‐*trn*L and *psb*A‐*trn*H) (see Johnson et al., 2017 for additional details of phylogenetic methods). The ultrametric tree was pruned to include only the 34 species used in this study using the *drop. tip* function in the R package ape (Paradis et al., 2004). For those species that included representatives from multiple islands, a single representative from the island sampled in this study was selected. Overall, the tree was well‐resolved with most major clades having support values ≥70 BS (maximum likelihood bootstrap support) and ≥0.95 PP (Bayesian posterior probability) (Figure 1).

### 2.4 Phylogenetic signal

Phylogenetic signal (the tendency for closely related species to resemble one another) was evaluated for each of the functional traits with the *multiphylosignal* function in the R package picante (Kembel et al., 2008), which uses generalized least squares to calculate Blomberg's *K* statistic (Blomberg et al., 2003). The species mean for each trait was used to calculate phylogenetic signal, except for height, in which the maximum for each species was used. Values of *K* < 1 indicate weak phylogenetic signal and suggest that closely related species are more different from one another than expected by chance (i.e., trait divergence). Values of *K* ≈ 1 approach a Brownian motion model of trait evolution, where traits change by small random amounts and at a constant rate through time. Values of *K* > 1 indicate strong phylogenetic signal and suggest that closely related species are more similar than expected by chance (i.e., trait conservatism). Given that picante only tests for significant differences from zero, the *phylosig* function in the R package phytools (Revell, 2012) was used to test for significant differences from one by conducting randomization tests.

### 2.5 Phylogenetic community structure

Phylogenetic structure was estimated at two spatial scales: (1) island communities (regional spatial scale) and (2) site communities within islands (local spatial scale). To estimate phylogenetic structure at both scales, the R package picante (Kembel et al., 2008) was used to calculate mean pairwise distance (MPD) and mean nearest taxon distance (MNTD; Webb, 2000). MPD is a measure of tree‐wide clustering versus overdispersion, while MNTD is a measure of clustering versus overdispersion at the branch tips. The ultrametric phylogeny of 34 species was converted into a distance matrix using the *cophenetic* function in the R stats package, which computes the pairwise distances between tips using branch lengths. Null models were then used to compare both the MPD and the MNTD metrics to expectations under neutral theory (Hubbell, 2001). Null communities were generated using the independent swap null algorithm, in which the number of species in each community and the frequency of occurrence of each species across communities were held constant, while the species that coexist in each community were randomized (Gotelli & Entsminger, 2003). Incorporating these assumptions into the null model has been shown to be effective in minimizing Type I error (Kembel & Hubbell, 2006). Standardized effect sizes (SES) of MPD and MNTD were calculated across 1000 null communities. A negative SES value indicates clustering while a positive value indicates overdispersion (Webb et al., 2002). The number of communities with negative and positive SES values was counted for both metrics. Statistical significance was determined by estimating *p* values based on the proportion of simulated means that were more (clustered) or less (overdispersed) extreme than the observed means (*α* = 0.05). Phylogenetic beta diversity (i.e., intercommunity structure) between islands was also calculated using the MNTD metric with the *comdisnt* function in picante (Kembel et al., 2008).

### 2.6 Trait‐based community structure

As with phylogenetic structure, trait structure was estimated for islands (regional spatial scale) and sites within islands (local spatial scale). The functional trait structure of both community types was also assessed with the MPD and MNTD metrics, with trait distance replacing phylogenetic distance. Analyses were conducted on both individual traits and on all 10 traits combined, using the mean value (or maximum for height) of each trait. Mean trait values were log10‐transformed as needed and scaled to improve normality. Trait statistics were calculated by using species presence–absence data for each community. The SES of MPD and MNTD were calculated across 1000 null communities using the independent swap algorithm. Clustered and overdispersed communities were determined as for phylogenetic community structure. Interisland community trait structure was also calculated using the MNTD metric as above.

## 3 RESULTS

### 3.1 Study sites and species

A total of 276 individuals were sampled from 34 species of *Cyrtandra* across 19 sites on 6 islands (Table 1). Sampled elevations ranged from 20 to 1270 m, and included four habitat types: lowland rainforest, montane rainforest, montane mesic forest, and cloud forest (sensu Mueller‐Dombois & Fosberg, 1998). The mean number of observed *Cyrtandra* species per site was 3 (min = 1; max = 5), and the mean number of individuals sampled per site was 15 (min = 3; max = 48).

### 3.2 Functional traits

**FIGURE 2:**
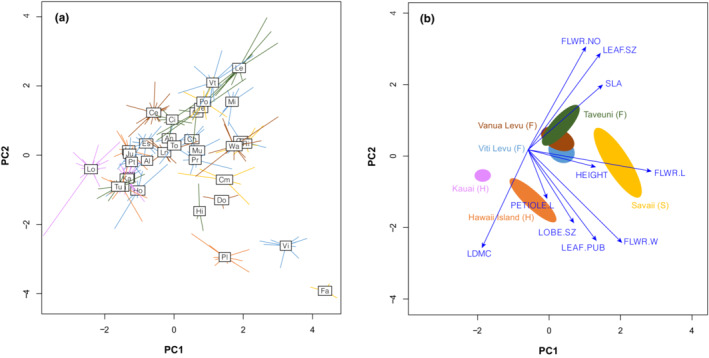
Results from a principal components analysis including 10 functional traits. The first two components (PC1 and PC2) represent 45% of the variation. The distributions of 34 Pacific *Cyrtandra* species (see species codes in Table S2) are shown in functional trait space (a) and each of the six sampled islands from three archipelagos (F—Fiji, S—Samoa, H—Hawaii) are represented in trait space with 95% confidence ellipses; arrows represent the contribution of each trait to functional phenotype (b).

Across all 34 species examined, maximum plant height ranged from sprawling shrubs as low as 115 cm (e.g., *C. hispida*) to small trees as tall as 6 m (e.g., *C. richii*) (Table S2). Mean values for leaf traits varied across all species as follows (abbreviations as in Table 2): SLA 14–41 m^2^ kg^−1^, LDMC 79–224 mg g^−1^, petiole length 2–14 cm, leaf size 29–776 cm^2^, leaf pubescence 0–362 trichomes per cm^2^. Mean values for floral traits varied across all species as follows: flower length 15–46 mm, flower width 5–14 mm, corolla lobe size 22–570 mm^2^, and floral display size 1–15 flowers (Table S2). Within species, most traits exhibited low levels of variation (Table S2; Figure 2a), although high intraspecific variation was observed in height and leaf size (Table S2). The PCA explained 71% of the variation across the first four axes. The first PCA axis was largely defined by corolla tube length and width followed by SLA and leaf size (26% of the variation; Figure 2b; Table 3); the second PCA axis was defined by flower number, LDMC, leaf size, and leaf pubescence (19% of the variation; Figure 2b; Table 3); the third PCA axis was defined by petiole length, corolla lobe size, flower number, and SLA (15% of the variation; Table 3); and the fourth PCA axis was defined by height, LDMC, and leaf pubescence (11% of the variation; Table 3).

**TABLE 3:** Principal components analysis (PCA) loadings for 10 functional traits across the first four axes (71% of variation). The strongest contributors to each principal component are in bold (based on a cutoff of $\sqrt{\left(1/10\right)}$ = ±0.316).

Functional trait	PC1	PC2	PC3	PC4
Corolla tube length	**0.511**	–	0.259	–
Corolla tube width	**0.412**	**−0.374**	−0.169	−0.249
Leaf size	**0.325**	**0.400**	−0.261	−0.120
Specific leaf area	**0.325**	0.259	**0.321**	0.161
Flower number	0.265	**0.432**	**−0.339**	–
Leaf dry matter content	−0.214	**−0.416**	−0.123	**−0.464**
Leaf pubescence	0.307	**−0.372**	−0.274	**0.355**
Petiole length	–	−0.210	**−0.528**	0.224
Corolla lobe size	0.247	−0.283	**0.499**	–
Maximum height	0.281	–	–	**−0.744**

### 3.3 Phylogenetic signal

Estimates of phylogenetic signal in the 10 functional traits resulted in a range of *K* values, from 0.34 for petiole length to 0.89 for flower width (Table 4). Five traits (SLA, LDMC, petiole length, leaf pubescence, and maximum height) had *K* values that were significantly different from one, suggesting the absence of phylogenetic signal in these traits. In contrast, four traits (leaf size, flower length, flower width, and flower number) had *K* values significantly different from zero, indicating the presence of phylogenetic signal. Corolla lobe size was not significantly different from one or zero, although there was a trend toward this trait exhibiting phylogenetic signal (*K* = 0.58, *p* = .09).

**TABLE 4:** Estimates of phylogenetic signal in functional traits using Blomberg's *K* (Blomberg et al., 2003).

Trait	Blomberg's *K*	Different from 0	Different from 1
Maximum height	0.45	0.233 NS	0.037*
SLA	0.50	0.150 NS	0.046*
LDMC	0.40	0.285 NS	0.013*
Petiole length	0.34	0.682 NS	0.008**
Leaf pubescence	0.36	0.603 NS	0.006**
Leaf size	0.67	0.003**	0.292 NS
Corolla tube length	0.63	0.003**	0.217 NS
Corolla tube width	0.89	0.025*	0.763 NS
Corolla lobe size	0.58	0.089NS	0.148 NS
Flower number	0.63	0.006**	0.236 NS

*Note*: Values of *K* < 1 indicate trait divergence, *K* ≈ 1 indicates random trait evolution (Brownian motion), and values of *K* > 1 indicate trait conservatism. Significant differences from the null expectation are indicated by the following: *p < .05, **p < .01; NS indicates a result not significantly different from the null expectation.

### 3.4 Phylogenetic community structure

*Cyrtandra* species assemblages on all six islands were phylogenetically clustered; four were significantly more clustered than null communities under the MPD metric (Viti Levu, Vanua Levu, Kaua‘i, and Hawai‘i Island; mean *Z*_score_ = −2.75, *p* = .007), and/or the MNTD metric (Viti Levu, Vanua Levu, Savaiʻi, and Hawaiʻi Island; mean *Z*_score_ = −2.53, *p* = .02) (Table S3). The Fijian Island of Taveuni was the only island that did not exhibit significant clustering as estimated by both metrics (MPD: *Z*_score_ = −1.22, *p* = .14; MNTD: *Z*_score_ = −1.11, *p* = .14). Interisland community structure inferred from the MNTD metric revealed several patterns (Table S4). The smallest phylogenetic distances were between island communities within the same archipelago. Within the Fijian archipelago, Viti Levu and Vanua Levu were most similar to Taveuni (geologically the youngest, and also the smallest island of the three), while Taveuni was most similar to the neighboring island of Vanua Levu. Between archipelagos, phylogenetic distances were smallest between island communities that were separated by the shortest geographic distance (e.g., Savaiʻi, Samoa was most similar to Taveuni, Fiji; Kauaʻi and Hawaiʻi Island were most similar to Viti Levu, Fiji).

Within islands, a total of 18 sites were examined for phylogenetic structure, with one site from Vanua Levu omitted due to the presence of only a single species at the site (*C. longifructosa*). Of the 18 sites, 15 were clustered and 3 were overdispersed (Table S5). Only four sites were significantly clustered relative to null communities under the MPD metric (Komave, Waisali, Voma, and Kokeʻe; mean *Z*_score_ = −1.67, *p* = .03), while all other sites were not significantly different from the null expectation (mean *Z*_score_ = −0.74, *p* = .27). Similar results were obtained under the MNTD metric, with 16 clustered sites and two overdispersed sites. Four communities were significantly clustered (Komave, Waisali, Matavanu, and Tomanivi; mean *Z*_score_ = −1.75, *p* = .03), while all others did not vary from the null expectation (mean *Z*_score_ = −0.79, *p* = .25).

### 3.5 Trait‐based community structure

Analyses of island community structure based on the combination of all 10 functional traits revealed that five islands were phenotypically clustered and one was overdispersed (Hawaiʻi Island), although none of the communities were significantly different from the null expectation under the MPD metric (mean *Z*_score_ = −0.54, *p* = .32) or the MNTD metric (mean *Z*_score_ = −0.57, *p* = .31) (Table S6). Island community structure based on individual traits suggested that four islands were significantly clustered for one or two traits under the MPD metric (Viti Levu, Vanua Levu, Savaiʻi, Kauaʻi). Clustered traits included maximum height, SLA, flower length, corolla lobe size, and flower number. In contrast, two islands were not significantly different from null communities in any of the traits examined (Taveuni Island and Hawaiʻi Island, the youngest islands in each respective archipelago). Under the MNTD metric, two islands were significantly clustered for maximum height (Vanua Levu) or flower length (Kauaʻi), while all others did not differ significantly from the null. Interisland community structure based on functional trait distances corroborated the patterns seen in the phylogenetic intercommunity analysis, with the single exception of Taveuni communities being most phenotypically similar to Viti Levu communities as opposed to Vanua Levu in the phylogenetic analysis (Table S7).

The community structure of sites within islands based on all functional traits combined revealed 10 clustered sites and eight overdispersed sites under the MPD metric (Table S8). Only three sites exhibited significant clustering (Colo‐i‐Suva, Korobaba, and Lavena; mean *Z*_score_ = −2.09, *p* = .03), while all other sites did not differ from null communities (MPD mean *Z*_score_ = 0.08, *p* = .52). Similar results were seen using the MNTD metric, with 10 clustered sites and eight overdispersed sites. Only one site was significantly clustered (Lavena, *Z*_score_ = −2.51, *p* = .03), while all other sites did not vary from the null (MNTD mean *Z*_score_ = −0.08, *p* = .48). Analyses based on individual traits revealed six communities that were significantly clustered under the MPD metric (Komave, Waisali, Des Voeux, Aʻopo, Matavanu, and Kokeʻe), while all other sites did not differ significantly from null communities. The MNTD metric revealed similar results, with six communities being significantly clustered (Komave, Korayanitu, Waisali, Des Voeux, Aʻopo, and Kokeʻe), and all others not differing from the null expectation. Traits that exhibited significant clustering under one or both metrics included maximum height, SLA, LDMC, petiole length, flower length, and flower number.

## 4 DISCUSSION

The present study examined patterns of coexistence within Pacific Island *Cyrtandra* across two spatial scales. The author sought to determine whether species of these plants, which are important components of the wet forest understory across islands of the Pacific Basin, are randomly assembled within island and site communities with respect to phylogenetic relatedness and traits that are ecologically relevant. Using a combination of 10 functional traits and a well‐resolved species phylogeny, the author reports evidence for nonrandom assembly within Pacific *Cyrtandra*.

### 4.1 Long‐distance dispersal followed by in situ diversification and hybridization

Coexisting species of *Cyrtandra* were generally found to be more closely related than expected by chance. This pattern was observed at both regional (island) and local (site) spatial scales. The trend of phylogenetic clustering observed in Pacific *Cyrtandra* aligns with findings presented by Johnson et al. (2019), which suggested a single colonization of the Hawaiian archipelago followed by multiple interisland dispersal events and varying levels of in situ diversification and hybridization among colonizing lineages. Once *Cyrtandra* arrived on distant Pacific archipelagos (likely via avian frugivory), available niche space and ample resources in the newly formed wet forest understories would have allowed small populations of closely related plants to persist without intense competition. These small populations likely remained isolated for many years, with genetic and morphological differences building up over time and resulting in situ diversification within islands. Subsequent dispersal events within and between islands, as well as cycles of range expansion and contraction following climatic shifts and ongoing volcanic eruptions, may have resulted in previously isolated populations coming together in zones of secondary contact. In these contact zones, hybridization may have contributed to increased genetic diversity in some lineages (Johnson et al., 2019) while driving the formation of reproductive barriers in others (Johnson et al., 2015). This interaction of processes over evolutionary timescales would have resulted in communities comprised of multiple closely related *Cyrtandra* species persisting in low densities as understory shrubs or small trees in wet forest environments.

### 4.2 Absence of trait conservatism

Examination of phylogenetic signal revealed *K* values of less than one for all 10 functional traits, suggesting that none of the traits examined are phylogenetically conserved among species. This result also suggests that closely related species resemble each other less than expected under a Brownian motion model of trait evolution, pointing to a role for selection rather than drift in shaping species assemblages. There was no evidence for phylogenetic signal in four of the five leaf traits examined (SLA, LDMC, petiole length, and leaf pubescence), whereas three of the four floral traits (flower length, flower width, and flower number) exhibited significant phylogenetic signal. Overall, this suggests that leaf traits tend to be more evolutionarily labile than floral traits in Pacific *Cyrtandra*.

### 4.3 Pollinators drive floral similarities among coexisting species

Coexisting species were also more phenotypically similar than expected by chance, and this was more evident in floral traits than leaf traits. At a regional spatial scale, floral traits that were clustered in most of the island communities included flower number (75%) and flower width (67%). At the local spatial scale, floral traits that were clustered within most site communities included flower length (69%) and flower width (61%). The retention of phylogenetic signal in three of the four floral traits examined and the strong influence of these traits on the observed degree of phylogenetic clustering may indicate that pollinators act as a strong biotic filter to establishment, driving similarities in floral morphology among coexisting species of *Cyrtandra*.

While some variation is seen in tube length, tube width, petal size, and floral display in Pacific taxa, this is minimal compared with the floral diversity described in Borneo, Indonesia, New Guinea, and the Philippines (Atkins et al., 2020). The floral variation seen among Southeast Asia *Cyrtandra* taxa suggests an array of pollinators including birds, bees, moths, and other flying insects. In contrast, species across the Pacific Basin are almost exclusively white‐flowered (a few have yellow or green flowers) suggesting pollination by small generalist insects, which may be an advantage to colonizing lineages (Cronk et al., 2005). The high degree of interspecific hybridization documented in Pacific *Cyrtandra*, particularly in the remote Hawaiian archipelago (Johnson et al., 2015, 2019; Kleinkopf et al., 2019; Pillon et al., 2013; Wagner et al., 1990), also supports the theory of a generalist pollinator. Although few observations of *Cyrtandra* pollinators exist, those that have been documented indicate nocturnal moths that feed on the nectar (Gardener & Daehler, 2006; Roelofs, 1979). Several characteristics typically displayed by island *Cyrtandra* seem to fit the pollinator syndrome of a small nocturnal flying insect: low plant density, sprawling plant habit, indistinct floral displays, flowers often hidden among the foliage or near the ground and containing large amounts of dilute nectar (M. Johnson, pers. obs.). While further research is needed to better understand the role of pollinators in Pacific *Cyrtandra* evolution, findings from the present study suggest that diversification in floral traits is constrained by a low diversity of generalist insect pollinators on islands.

### 4.4 Ecological specialization in rainforest understory habitats

Despite Pacific *Cyrtandra*'s propensity for dispersal across long distances, many taxa have restricted distributions within islands, suggesting that environmental conditions may act as a filter to establishment (Carlquist, 1965). The availability of wet forest understories seems a critical requirement for *Cyrtandra*, as few taxa are found outside this habitat (Cronk et al., 2005). Leaf size was clustered in 83% of island communities, while maximum height and leaf size were clustered in 64% and 61% of site communities, respectively. Plant height and leaf size are involved in water transport and light interception, two features that are key to establishment and survival in a rainforest understory environment. The contribution of these traits to the observed pattern of clustering, and the phylogenetic signal observed in leaf size at both regional and local scales may suggest that these traits are involved in the habitat‐specific physiological processes that exclude (filter) species that do not match environmental conditions. Environmental filtering may limit the divergent effects of resource competition, particularly in low‐density populations (such as those commonly observed in *Cyrtandra*) and promote the coexistence of closely related species with similar traits (Johnson et al., 2019).

The importance of similar environmental conditions between source areas and colonization sites is increasingly being recognized for island floras, with less emphasis being placed on dispersal limitation as the sole determining factor in nonrandom community assembly (Nathan, 2006). A study of the Galapagos flora by Carvajal‐Endara et al. (2017) suggested that habitat filtering was more important than dispersal limitation, as given enough time (e.g., millions of years) even poor dispersers may have an opportunity for colonization, while specific niche requirements may represent an insurmountable barrier to establishment. The evolution of fleshy fruits (and the association with avian frugivores) in New Guinea *Cyrtandra* species enabled these plants to overcome the open‐ocean barrier that limits all other genera of Gesneriaceae from dispersing east of the Solomon Islands (Burtt, 2001), and this key innovation likely contributed to the group's accelerated diversification across the Pacific (Roalson & Roberts, 2016). Cronk et al. (2005) described Pacific *Cyrtandra* as a “supertramp” clade (sensu Diamond, 1974) due to its high dispersability, which could compensate for the apparent high level of ecological specialization that might otherwise limit its expansion. The biogeographic analyses conducted by Johnson et al. (2017) supports this notion of Pacific *Cyrtandra* being highly dispersable, with 18 founder events recovered between Pacific archipelagos and 10 founder events recovered between islands within the Hawaiian archipelago. The requirement for wet shady forest conditions (like those inhabited by taxa in the source areas of Southeast Asia) likely prohibited colonizing taxa from becoming established on small Pacific islands that are predominantly arid (Ni‘ihau and Kaho‘olawe in Hawaii and Qamea in Fiji).

### 4.5 Potential limitations

The biogeographic scale at which studies of community assembly are conducted have been shown to be important regarding inferred dispersal limitations (Hardy et al., 2012), appropriate sampling across regional species pools (Kraft et al., 2007), and the influence of environmental gradients (Kraft et al., 2008). Concerning dispersal limitation, the approach used in the present study assumed that all species could disperse everywhere based on results from several studies examining biogeographic patterns within Pacific *Cyrtandra* (Clark et al., 2009; Johnson et al., 2017, 2019). However, Johnson et al. (2017) also suggested that dispersal between geographically distant islands is less frequent than between islands that are in proximity. Thus, a potential caveat of the present study is that our results may overestimate dispersal potential, and limiting sampling to regional species pools (i.e., within archipelagos) may produce slightly different results.

The results presented here may also be influenced by the sampling intensity of regional species pools. Simulation‐based analyses have revealed that studies including 30%–60% of the regional species diversity have the greatest statistical power to detect phylogenetic community structure (Kraft et al., 2007). In the present study, sampling percentages were 60% of Fijian *Cyrtandra* species, 21% of Samoan species, and 8% of Hawaiian species. Thus, inferences of phylogenetic community structure in Samoan and Hawaiian lineages may not be robust to issues related to sampling intensity. Increased sampling efforts in these two regions that contain high numbers of endemic *Cyrtandra* species is therefore needed to improve resolution in these analyses.

Lastly, patterns of assembly may be influenced by abiotic and biotic factors that change across climatic (Cavender‐Bares et al., 2006), edaphic (Fine & Kembel, 2011), and topographic (Kraft et al., 2008) gradients. To capture variation associated with habitats, the present study employed sampling strategies that were aimed at encompassing the full range of habitat types that Pacific *Cyrtandra* species occupy. Specifically, sites varied in elevation (20–1270 m), substrate age (0.5–29 million years ago), and vegetation type (lowland wet forest, montane mesic forest, montane wet forest, and cloud forest). Despite sampling across these broad gradients, there was no clear evidence of assembly structure that was related to any habitat, although further studies with more explicit sampling schemes may reveal trait responses that are linked to environmental variables (e.g., light, soil moisture; see Sedio et al., 2012) and/or topographic features (elevation, slope; see Lasky et al., 2014).

## 5 CONCLUSIONS

This study suggests that communities of Pacific *Cyrtandra* species are not randomly assembled, and instead, that niche‐based processes structure biodiversity at regional and local spatial scales in diverse congeneric species assemblages. The observation of phylogenetic and phenotypic clustering within island and site communities suggests that coexisting species are more closely related than expected by chance and that trait‐based patterns are reflective of evolutionary relatedness at both spatial scales. The finding of significant clustering at the local spatial scale is in contrast with findings from many other plant communities that report phylogenetic and functional trait overdispersion due to resource competition at this small spatial scale. This may be related to reduced competition in these habitats due to low population densities of *Cyrtandra* taxa and/or higher resource availability in oceanic island rainforest understories that are species poor due to dispersal limitations of many taxa. The floral trait similarities observed among closely related coexisting species may be driven by generalist pollinators on oceanic islands, while a high degree of ecological specialization in rainforest understories may explain the similarity in leaf size and height among coexisting taxa. As with most other recently published comparable studies, these results may be highly contingent on the choice of metrics used to assess community structure, the set of communities, and on the selection of functional traits. Studies that increase regional sampling in areas with high species diversity and incorporate estimates of abiotic variables associated with microhabitats would be needed to further address the underlying drivers of assembly patterns described here.

## AUTHOR CONTRIBUTIONS

**Melissa A. Johnson:** Conceptualization (equal); data curation (equal); formal analysis (equal); funding acquisition (equal); investigation (equal); methodology (equal); project administration (equal); resources (equal); software (equal); supervision (equal); validation (equal); visualization (equal); writing – original draft (equal); writing – review and editing (equal).

## CONFLICT OF INTEREST STATEMENT

None.

## Supporting information


Table S1.

Table S2.

## Data Availability

The data that support the findings of this study are included in the supplementary materials.
