# Supplementary material for: Phylogenetic and functional trait‐based community assembly within Pacific Cyrtandra (Gesneriaceae): Evidence for clustering at multiple spatial scales
Source: Ecol Evol. 2023 May 4;13(5):e10048. doi: 10.1002/ece3.10048 (PMC10160169; doi:10.1002/ece3.10048)
Supplement: Supplementary file 1 — Table S1. [file ECE3-13-e10048-s002.docx]

**Supplementary Table S1.** Description of Pacific Islands sampled for *Cyrtandra*.

| **Island** | **Country** | ***Cyrtandra* Species Richness** | **Species Sampled (No.)** | **Maximum Age (MY)** | **Land Area**  **(km^2^)** | **Highest Elevation (m)** | **Distance to Nearest Major Island (km)** | **Distance to Nearest Continent (km)** |
| --- | --- | --- | --- | --- | --- | --- | --- | --- |
| Viti Levu | Fiji | 25 | 18 | 29.0 | 10388 | 1323 | 60 | 2630 |
| Vanua Levu | Fiji | 13 | 4 | 8.0 | 5535 | 1032 | 60 | 2630 |
| Taveuni | Fiji | 9 | 6 | 3.0 | 434 | 1241 | 9 | 2630 |
| Savaiʻi | Samoa | 12 | 4 | 5.2 | 1820 | 1860 | 18 | 3800 |
| Kauaʻi | Hawaii | 17 | 2 | 4.7 | 1574 | 1569 | 29 | 3765 |
| Hawaiʻi Island | Hawaii | 11 | 3 | 0.5 | 10430 | 4205 | 42 | 3765 |

**Supplementary Table S3.** Island phylogenetic structure based on standardized effect sizes (SES) of mean pairwise distance (MPD) and mean nearest taxon distance (MNTD). Islands are listed in order of increasing species richness (SR). The ‘−’ sign indicates phylogenetic clustering, while the ‘+’ sign indicates phylogenetic over-dispersion. Significant differences from the null model are indicated by the following: ‘*’ p < 0.05, ‘**’ p < 0.01, ‘***’ p < 0.001.

| **Community** | **Country** | **SR** | **SES.MPD** | **SES.MNTD** |
| --- | --- | --- | --- | --- |
| Kaua’i | Hawaii | 3 | − (**) | − |
| Hawai’i Is. | Hawaii | 3 | − (*) | − (*) |
| Savai’i | Samoa | 4 | − | − (**) |
| Vanua Levu | Fiji | 5 | − (**) | − (*) |
| Taveuni | Fiji | 6 | − | − |
| Viti Levu | Fiji | 17 | − (***) | − (***) |

**Supplementary Table S4.** Community phylogenetic structure based on standardized effect sizes (SES) of mean pairwise distance (MPD) and mean nearest taxon distance (MNTD). Communities are listed in order of increasing species richness (SR). The ‘−’ sign indicates phylogenetic clustering, while the ‘+’ sign indicates phylogenetic over-dispersion. Significant differences from the null model are indicated by the following: ‘*’ p < 0.05, ‘**’ p < 0.01.

| **Community** | **Country** | **SR** | **SES.MPD** | **SES.MNTD** |
| --- | --- | --- | --- | --- |
| A’opo | Samoa | 2 | + | + |
| Komave | Fiji | 2 | − (*) | − (**) |
| Taga | Samoa | 2 | − | − |
| Koke’e State Park | Hawaii | 3 | − (*) | − |
| Lavena | Fiji | 3 | − | − |
| Nambukelevu | Fiji | 3 | − | − |
| Waiakea Forest Reserve | Hawaii | 3 | − | − |
| Waisali Forest Reserve | Fiji | 3 | − (*) | − (*) |
| Des Voeux Peak | Fiji | 4 | + | + |
| Matavanu Crater | Samoa | 4 | + | − (*) |
| Mt. Koroyanitu | Fiji | 4 | − | − |
| Navua River | Fiji | 4 | − | − |
| Mt. Voma | Fiji | 6 | − (*) | − |
| Colo-i-Suva | Fiji | 7 | − | − |
| Mt. Korobaba | Fiji | 7 | − | − |
| Mt. Lomalagi | Fiji | 7 | − | − |
| Mt. Naitaradamu | Fiji | 10 | − | − |
| Mt. Tomanivi | Fiji | 10 | − | − (*) |

**Supplementary Table S5.** Island community trait structure based on mean pairwise trait distance (MPD) and mean nearest trait distance (MNTD). The ‘−’ sign indicates phylogenetic clustering, while the ‘+’ sign indicates phylogenetic over-dispersion. Significant differences from the null model are indicated by the following: ‘*’ p < 0.05, ‘**’ p < 0.01.

| Community | Metric | Height | SLA | LDMC | Petiole.L | Leaf.Sz | Pubesc. | Flwr.L | Flwr.W | Lobe.Sz | Flwr.No | All traits |
| --- | --- | --- | --- | --- | --- | --- | --- | --- | --- | --- | --- | --- |
| Hawaii Is. | MPD  MNTD | +  + | −  − | +  + | +  + | +  + | +  + | −  − | +  + | −  − | −  − | +  + |
| Kauai | MPD  MNTD | +  + | −  − | −  − | −  − | −  − | +  − | − (*)  − (*) | −  − | +  − | − (*)  − | −  − |
| Savaii | MPD  MNTD | +  − | − (*)  − | −  − | +  + | −  − | +  + | −  + | −  − | +  + | −  − | −  − |
| Vanua Levu | MPD  MNTD | − (*)  − (*) | +  + | −  − | −  − | −  − | −  − | +  + | −  − | +  − | +  − | −  − |
| Taveuni | MPD  MNTD | +  − | +  + | +  + | −  − | −  − | −  − | −  + | −  − | +  + | +  − | −  − |
| Viti Levu | MPD  MNTD | − (*)  − | +  + | +  + | −  + | −  − | −  + | +  + | +  + | − (*)  − | −  + | −  − |

**Supplementary Table S6.** Community trait structure based on standardized effect sizes (SES) of mean pairwise trait distance (MPD) and mean nearest trait distance (MNTD) for individual traits, and for all 10 functional traits combined. The ‘−’ sign indicates phylogenetic clustering, while the ‘+’ sign indicates phylogenetic over-dispersion. NA refers to a lack of data for at least one species, such that pairwise comparisons were not possible. Significant differences from the null model are indicated by the following: ‘*’ p < 0.05, ‘**’ p < 0.01, ‘***’ p < 0.001.

| Community | Metric | Height | SLA | LDMC | Petiole.L | Leaf.Sz | Pubesc. | Flwr.L | Flwr.W | Lobe.Sz | Flwr.No | All traits |
| --- | --- | --- | --- | --- | --- | --- | --- | --- | --- | --- | --- | --- |
| A’opo | MPD  MNTD | − (*)  − (*) | −  − | − (*)  − (*) | −  − | −  − | −  − | −  − | −  − | +  + | +  + | −  − |
| Komave | MPD  MNTD | +  + | −  − | −  − | − (**)  − (*) | +  + | +  + | +  + | NA  NA | NA  NA | −  − | −  − |
| Taga | MPD  MNTD | +  + | −  − | −  − | −  − | −  − | −  − | −  − | +  + | +  + | −  − | −  − |
| Koke’e State Park | MPD  MNTD | +  + | +  + | −  − | −  − | −  − | +  + | − (*)  − (*) | −  − | +  + | − (**)  − (*) | −  − |
| Lavena | MPD  MNTD | −  − | −  − | −  − | −  − | −  − | −  − | −  − | −  − | −  − | −  − | − (***)  − (**) |
| Nambukelevu | MPD  MNTD | +  + | +  + | +  + | −  − | +  + | +  + | +  + | −  − | +  + | +  + | +  + |
| Waiakea Forest Reserve | MPD  MNTD | +  + | −  − | +  + | +  + | +  + | +  + | −  − | +  + | −  − | −  + | +  + |
| Waisali Forest Reserve | MPD  MNTD | − (*)  − (*) | +  + | −  + | −  − | −  + | −  − | +  + | −  − | +  + | +  + | +  + |
| Des Voeux Peak | MPD  MNTD | +  + | +  + | +  + | − (*)  − (**) | −  − | +  + | +  + | −  − | +  + | +  + | +  + |
| Matavanu Crater | MPD  MNTD | +  − | − (*)  − | −  − | −  − | −  − | +  + | −  + | −  − | +  + | −  − | +  − |
| Mt. Koroyanitu | MPD  MNTD | −  − (*) | −  − | +  + | +  + | +  − | −  − | −  − | −  − | −  − | +  + | +  + |
| Navua River | MPD  MNTD | −  − | −  − | +  + | +  + | +  + | +  + | −  − | −  + | −  − | +  + | +  + |
| Mt. Voma | MPD  MNTD | −  − | −  − | −  + | +  + | +  + | +  + | −  − | +  + | −  − | +  + | −  + |
| Colo-i-Suva | MPD  MNTD | −  − | +  + | −  − | +  + | −  − | −  − | −  + | −  − | −  − | −  − | − (*)  − |
| Mt. Korobaba | MPD  MNTD | −  − | +  + | −  − | +  + | −  − | −  − | −  + | −  − | −  − | −  − | − (*)  − |
| Mt. Lomalagi | MPD  MNTD | −  − | −  − | +  + | −  − | +  + | +  + | −  − | +  + | −  − | +  + | +  − |
| Mt. Naitaradamu | MPD  MNTD | −  − | +  + | −  − | +  + | −  − | −  − | −  − | −  + | −  − | −  − | −  + |
| Mt. Tomanivi | MPD  MNTD | −  − | +  − | −  + | +  + | −  − | +  + | −  − | +  + | −  − | −  − | −  − |

**Supplementary Table S7.** Inter-island community phylogenetic distance based on mean nearest taxon distance (MNTD).

|  | **Viti Levu** | **Vanua Levu** | **Taveuni** | **Savai’i** | **Kaua’i** |
| --- | --- | --- | --- | --- | --- |
| **Vanua Levu** | 11.304 |  |  |  |  |
| **Taveuni** | 9.901 | 8.229 |  |  |  |
| **Savai’i** | 16.042 | 15.863 | 13.399 |  |  |
| **Kaua’i** | 21.255 | 21.667 | 21.667 | 21.667 |  |
| **Hawai’i Is.** | 21.255 | 21.667 | 21.667 | 21.667 | 5.665 |

**Supplementary Table S8.** Inter-island community trait distance based on log10-transformed trait values and mean nearest Euclidean trait distance (MNTD).

|  | **Viti Levu** | **Vanua Levu** | **Taveuni** | **Savai’i** | **Kaua’i** |
| --- | --- | --- | --- | --- | --- |
| **Vanua Levu** | 2.728 |  |  |  |  |
| **Taveuni** | 2.460 | 2.480 |  |  |  |
| **Savai’i** | 3.281 | 3.167 | 3.049 |  |  |
| **Kaua’i** | 3.717 | 4.071 | 3.927 | 4.925 |  |
| **Hawai’i Is.** | 3.497 | 3.643 | 3.693 | 4.287 | 2.195 |
